# A lasered mouse model of retinal degeneration displays progressive outer retinal pathology providing insights into early geographic atrophy

**DOI:** 10.1038/s41598-019-43906-z

**Published:** 2019-05-16

**Authors:** Paul Ibbett, Srinivas V. Goverdhan, Elena Pipi, Joe K. Chouhan, Eloise Keeling, Elizabeth M. Angus, Jenny A. Scott, Maureen Gatherer, Anton Page, Jessica L. Teeling, Andrew J. Lotery, J. Arjuna Ratnayaka

**Affiliations:** 1 Biological Sciences, University of Southampton, SGH, South Lab and Path Block, MP840, Tremona Road, Southampton, SO16 6YD United Kingdom; 2 Clinical and Experimental Sciences, Faculty of Medicine, University of Southampton, MP806, Tremona Road, Southampton, SO16 6YD United Kingdom; 3 Eye Unit, University Hospital Southampton NHS Foundation Trust, Southampton, SO16 6YD United Kingdom; 4 Biomedical Imaging Unit, University of Southampton, MP12, Tremona Road, Southampton, SO16 6YD United Kingdom

**Keywords:** Diseases, Macular degeneration

## Abstract

Early stages of geographic atrophy (GA) age-related macular degeneration is characterised by the demise of photoreceptors, which precedes the loss of underlying retinal pigment epithelial (RPE) cells. Sight-loss due to GA has no effective treatment; reflecting both the complexity of the disease and the lack of suitable animal models for testing potential therapies. We report the development and characterisation of a laser-induced mouse model with early GA-like pathology. Retinas were lasered at adjacent sites using a 810 nm laser (1.9 J/spot), resulting in the development of confluent, hypopigmented central lesions with well-defined borders. Optical Coherence Tomography over 2-months showed progressive obliteration of photoreceptors with hyper-reflective outer plexiform and RPE/Bruch’s membrane (BrM) layers within lesions, but an unaffected inner retina. Light/electron microscopy after 3-months revealed lesions without photoreceptors, leaving the outer plexiform layer apposed to the RPE. We observed outer segment debris, hypo/hyperpigmented RPE, abnormal apical-basal RPE surfaces and BrM thickening. Lesions had wedge-shaped margins, extended zones of damage, activated Müller cells, microglial recruitment and functional retinal deficits. mRNA studies showed complement and inflammasome activation, microglial/macrophage phagocytosis and oxidative stress providing mechanistic insights into GA. We propose this mouse model as an attractive tool for early GA studies and drug-discovery.

## Introduction

**Figure 1:**
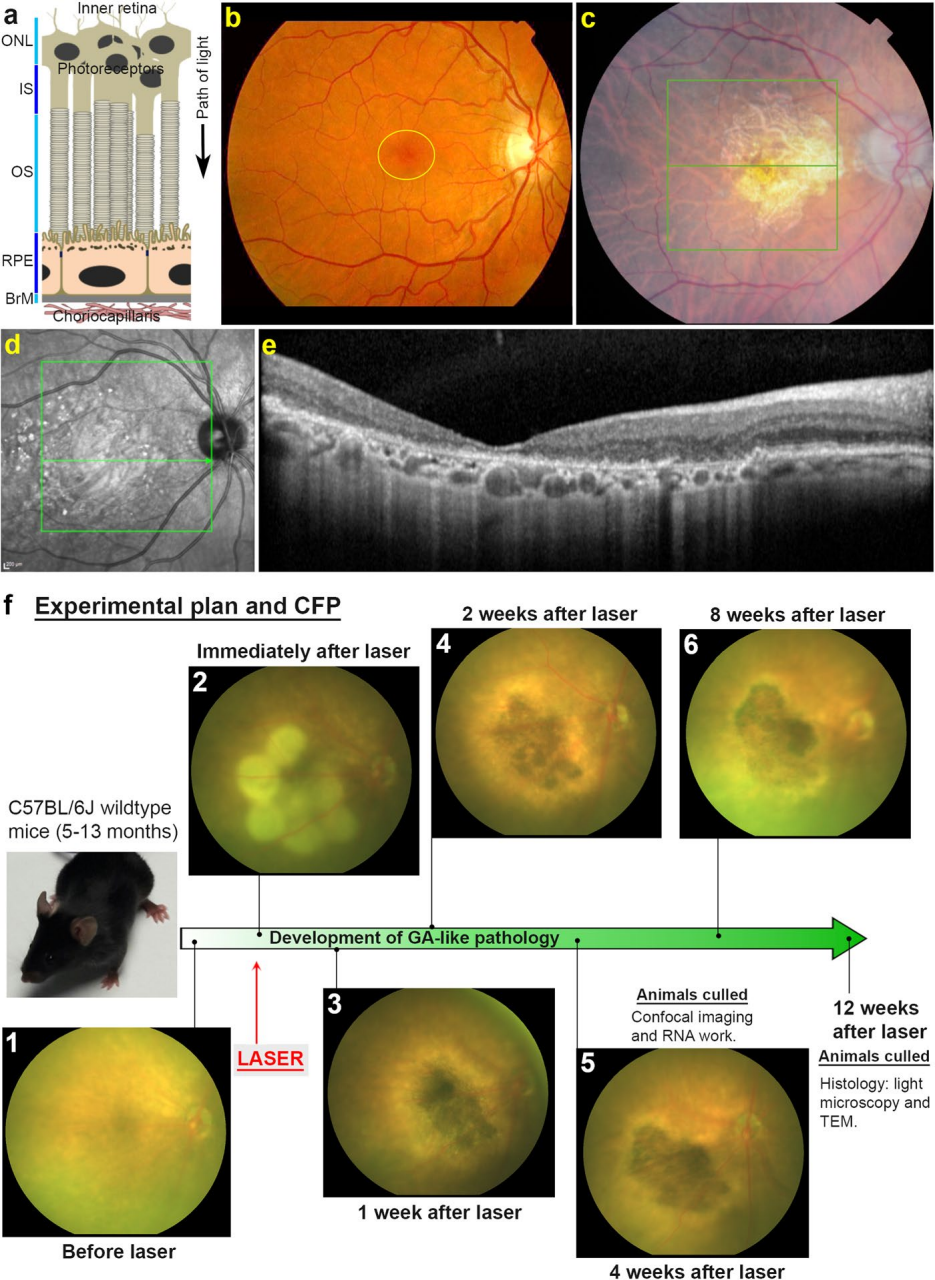
The anatomy of geographic atrophy (GA) and development of GA-like pathology in lasered mice. (**a**) Schematic diagram of the outer retina showing arrangement of different cell layers. Outer nuclear layer (ONL), Photoreceptor IS (inner segments) and OS (outer segments), Retinal pigment epithelium (RPE), Bruch’s membrane (BrM) and Choriocapillaris. An arrow indicates the path of light. (**b**) A colour fundus photograph (CFP) of a healthy human retina with the macula encircled in yellow, (**c**) compared to a diseased retina from a GA patient. Notice presence of a central/macular lesion with defined borders. (**d**) Black and white optical coherence tomography scan of a GA lesion taken from a Heidelberg OCT, and (**e**) a cross-section (from central green line in **d**) showing respective layers of the outer retina affected by disease. (**f**) Development of GA-like pathology in lasered mice. Representative CFP from mice before and after laser and in subsequent weeks. Notice how multiple lasered spots in the central murine retina coalesce after 1 week to create a focused atrophic region. Longitudinal CFP imaging of a five month old mouse shows this GA-like lesion to persist for up to 8 weeks after laser treatment. The timeline of developing GA-like pathology alongside experimental end-points are also shown.

Sight loss caused by damage to the central retina in age-related macular degeneration (AMD) represents the most common cause of irreversible blindness in developed societies. Globally, early stages are estimated to affect > 150 million individuals, whilst advanced AMD accounts for ~10 million patients^1^. AMD has a complex aetiology and initiates in the retinal pigment epithelium (RPE) and associated tissues of the outer retina. The RPE lies underneath the neuroretina and in intimate association with overlying photoreceptors. The RPE monolayer rests on a thin porous tissue termed Bruch’s membrane (BrM), underneath which the choriocapillaris delivers oxygen/nutrients to the outer retina whilst removing metabolic waste (Fig. 1a). Damage occurs to RPE cells over many years before changes become apparent in the form of extracellular protein/lipid deposits referred to as drusen. Their appearance in the macula, the central part of the retina responsible for high-acuity/focused vision, is the first clinical indicator of AMD, although patients do not necessarily report visual problems at this early stage. Other early indicators include pigmentary and structural RPE changes as well as retinal and choroidal thinning^2^. Advanced AMD is characterised by two broad phenotypes; geographic atrophy (GA/dry AMD) or neovascular AMD (nv/wet AMD), which occur at similar frequencies^3,4^. Histological studies of donor GA tissues show loss of photoreceptors early in disease possibly due to dysfunction of the underlying RPE^5–7^. These findings alongside more recent work confirm that in most patients, the demise of photoreceptors occur earlier than loss of RPE cells^8^. Currently, GA patients have no effective treatment whatsoever. Dietary supplementation remains the only means by which the odds of developing advanced AMD may be reduced, although this appears to be more effective against nvAMD^9^. In nvAMD, patients experience rapid loss of central vision associated with appearance of leaky/invasive choroidal vessels, breaks in the blood-retinal-barrier (BRB) and widespread retinal damage and scarring. Most nvAMD patients can be treated with intravitreal vascular endothelial growth factor (VEGF) inhibitors^10^, although prolonged treatment or chronicity of AMD can result in developing GA^11^.

A wide range of rodent, rabbit and non-human primate models have been used to study AMD, which offer advantages as well as limitations^12–14^. Mice for instance do not possess an anatomical macula nor reproduce the full disease spectrum, but still serves as a powerful tool for studies into AMD pathobiology and as a testbed for drug development. For example, development of the acute laser-induced nvAMD mouse model, although imperfect, enabled initial validation of anti-VEGF inhibitors^12^. In contrast, there is a lack of realistic and reproducible mouse models to study GA. Mice are attractive for such studies as they recapitulate salient GA-like features including focal photoreceptor atrophy, development of RPE abnormalities, lipofuscin, drusen-like deposits as well as BrM and choroidal pathology. Unfortunately, these phenotypes are not observed in a single mouse, but rather across several transgenic, acutely-induced or spontaneously arising mouse strains. Some of the more attractive mouse models can recapitulate features of both GA and nvAMD, but have to be aged before the desired phenotype manifests, even though they have been genetically modified to develop retinal pathology^12,13,15^. The penetrance of disease phenotypes in these models is also low, necessitating large cohorts to obtain useful animals in sufficient numbers. Furthermore, ocular pathology appears to manifest globally across the retina, rather than as an expanding geographic lesion in patients.

Here, for the first time, we describe the development and in-depth cellular and molecular characterisation of an acute onset early GA-like mouse model with progressive retinal pathology. Following laser exposure, adult C57BL/6J wildtype mice developed lesions that corresponded to areas with focal atrophic photoreceptors, abnormal RPE and BrM, inflammation as well as functional retinal defects indicative of progressive GA-like pathology. These features were observed in the absence of breaks in the BRB, neovascularisation or VEGF mRNA or protein upregulation, which are hallmarks of nvAMD. This GA-like mouse model appears to be markedly superior to existing alternatives and presents an altogether new tool to study disease pathways associated with early GA.

## Results

### Dose dependency between laser power and focal outer retinal pathology

To develop a mouse model with reproducible early GA-like features we exposed retinas to different intensities of the 810 nm diode laser at three different settings; henceforth referred to as low-powered (22 mW), medium-powered (32 mW) and high-powered (42 mW). In our initial optimisation study, an average of 7 laser spots were generated per mouse, with a duration of 60 seconds per spot (total energy per spot: low-powered, 1.3 J, medium-powered, 1.9 J and high-powered, 2.5 J). The effects were assessed by colour fundus photography (CFP) based on discolouration indicative of retinal atrophy (Supplementary Fig. S1). At 4 weeks post-treatment, the low-powered laser induced only minimal pathology. By contrast, mice subjected to medium or high-powered laser treatment developed visible atrophic lesions, characterised by focal hyperpigmentation surrounded by bright hazy margins. Using the medium-powered laser, we were able to create several small, localised areas of atrophy, which merged to form a recognisable GA-like lesion with well-defined borders (Supplementary Fig. S1). High-powered laser treatment resulted in a large-scale confluent and atrophic region. Use of optical coherence tomography (OCT) in eyes treated with low-powered laser showed little or no abnormalities in the retina, RPE/BrM or in the choroidal layer after 4 weeks, which was consistent with their CFP data (Supplementary Fig. S1). By contrast, OCT scans of mice treated with the medium-powered laser consistently developed hypo-reflective outer retinal pathology. OCT scans also showed the absence of photoreceptor inner/outer segments and the outer nuclear layer (ONL). Furthermore, the inner nuclear layer (INL) now appeared to lie in close apposition to the hyper-reflective areas of the lesion. Some distinct laser-treated spots could be discerned by OCT, while other lesions appeared to have coalesced after 4 weeks (Supplementary Fig. S1). OCT scans from mice treated with the high-powered laser mirrored CFP data, showing the rapid development of a large, confluent, hyper-reflective region. Hyper-reflective areas of such mice appeared to be extensive (wider and thicker) compared to mice treated with the medium-powered laser. We also observed the inner neuroretina of mice treated with the high-powered laser to be compromised after 4 weeks with discernible changes to the INL and inner plexiform layers (IPL) (Supplementary Fig. S1). It is worthwhile noting that even for the same laser power setting, we observed different levels of retinal discolouration at each spot after 60 seconds. This variation is most likely due to the need to slightly re-orient the animals’ position for each spot, which consequently required minor focal or optical adjustments. Considering this study as a whole, and observing the damage caused by different levels of discoloration, we opted to use the medium-power setting for each spot until the faintest burn could be clearly demarcated. While an average of 60 seconds was required to achieve a faint burn, the decision to focus on achieving consistency in discolouration instead of an overall set-time, produced a greater level of reproducibility for this model. Indeed, using this approach, we observed the development of lesions in 63/80 animals, yielding an overall success rate of 80%.

### Longitudinal retinal scans show development of GA-like lesions in lasered mice

**Figure 2:**
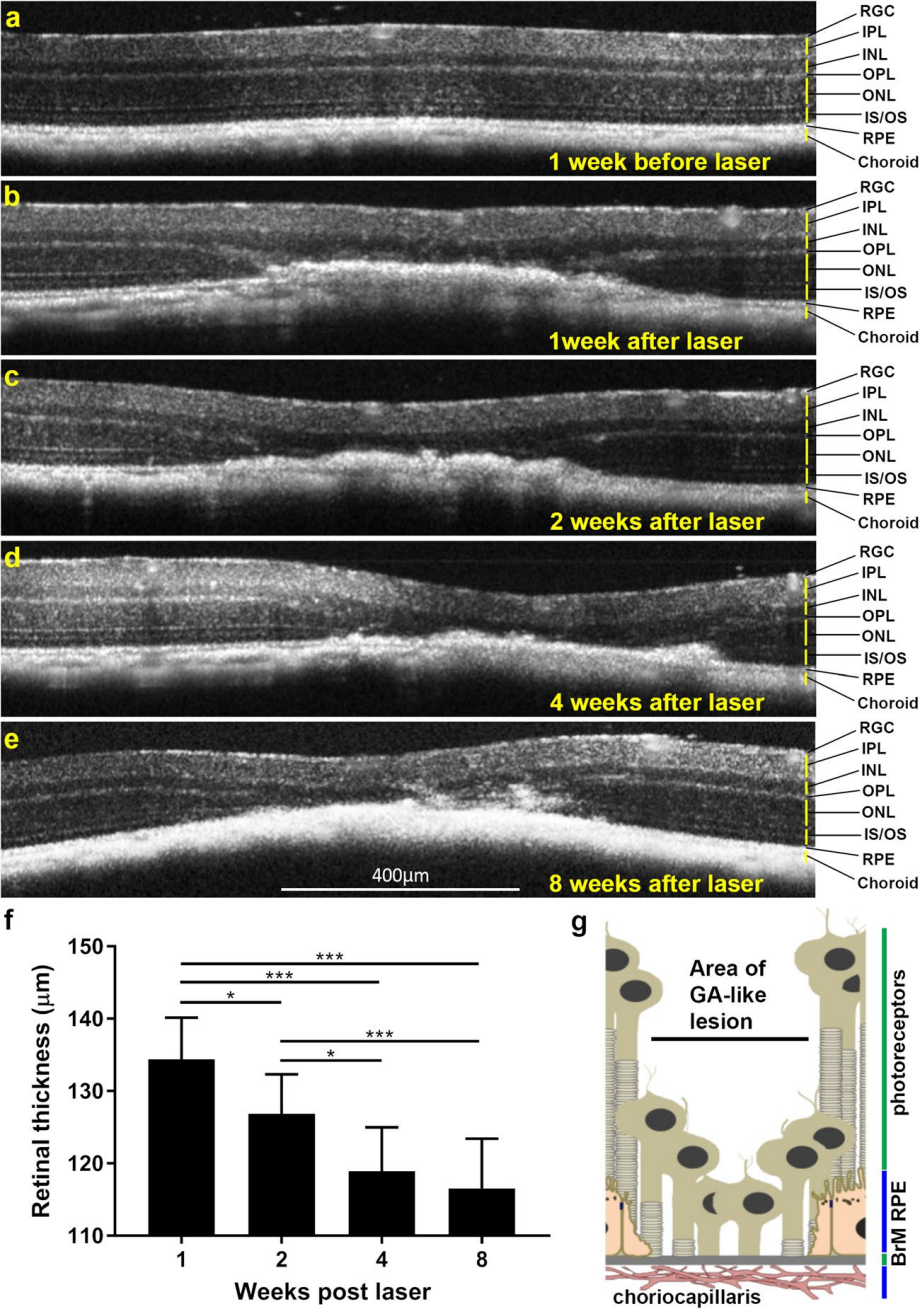
Longitudinal optical coherence tomography (OCT) of lasered mouse retinas show development of GA-like pathology. Representative OCT scan (**a**) 1 week before and (**b**) 1 week after laser treatment. Notice obliteration of the ONL and photoreceptor IS/OS. The clear demarcation of the RPE-BrM layer is also lost, although further details of pathology are difficult to distinguish. The OPL tapers into lesions and is no longer discernible as a distinct layer above the lasered spot. (**c**–**e**) Over the following weeks, lesions continue to persist, indicated by thickened areas of hyper-reflective pathology with progressive involvement of the INL and IPL, which appear to merge into lasered areas. Use of OCT scans thus provided insights into the involvement of distinct retinal layers over a 2 month period following laser treatment. Retinal ganglion cells (RGC), Inner plexiform layer (IPL), Inner nuclear layer (INL), Outer plexiform layer (OPL), Outer nuclear layer (ONL), IS/OS (inner and outer segments) of photoreceptors and Retinal pigment epithelium (RPE). OCT scans however do not allow the RPE and Bruch’s membrane (BrM) to be distinguished as separate layers. (**f**) OCT data was used to quantify the relative thickness of retinal lesions between weeks 1, 2, 4 and 8 following laser ablation. A statistically significant decrease in retinal thickness at lesion sites was observed between weeks 1, 2 and 4, after which the thickness of the retina became stable between weeks 4 and 8. Average of three measurements per lesion (n = 7, five month old mice). SEM with significance denoted by *p < 0.05 and ***p < 0.0001. [**g**] Speculative diagram based on OCT data showing arrangement of retina and associated tissues within lasered spots.

Having established the efficacy and reproducibility of using a medium-powered laser to generate lesions, we assessed the structural progression of lesions over 8 weeks by CFP and OCT scans. Pathology of human GA is provided for reference (Fig. 1b–e) alongside representative CFP scans from lasered mice (Fig. 1f). At 1 week post-treatment, multiple lasered spots had coalesced to form GA-like lesions with distinct borders. The surrounding tissues had a bright, hazy appearance, possibly indicating inflammation. Over the following weeks lesions became more clearly demarcated, with the centre of lesions becoming increasingly hyper-pigmented, whilst hazy bright margins became progressively confined to the edges. No lesions or other retinal abnormalities were observed in non-lasered eyes, which acted as an internal control. FA showed increased visibility of the choroidal vessels due to RPE atrophy at 8 weeks post-treatment, but with no evidence of fluid leakage from chordal vessels and a hyper-fluorescent marginal zone of the lesion (Supplementary Fig. S2), similar to GA patients. To gain further insights how lesions evolved, we performed longitudinal OCT scans over a 2 month period (Fig. 2a–e). At 1 week post-laser, hyper-reflective pathology in the outer retina was observed to be thick and pronounced indicating obliteration of the ONL and IS/OS. Hyper-reflectivity also affected the OPL and RPE-BrM compared to adjacent non-lasered tissue. Over the following weeks, areas of hyper-reflective pathology appeared to slowly regress along with progressive displacement of the inner retinal layers (INL, IPL) towards the BrM/choroid at lasered sites. Our data reveal a progressive reduction in the retinal thickness at the lesion site (F_1.785, 10.71_ = 35.65, p ≤ 0.0001), which is statistically significant between each successive quantification at weeks 1, 2 and 4. However, no statistical differences in lesion sizes were recorded between weeks 4 and 8. Mean value at 1 week = 134.4 μm ± 5.8 S.E.M, 2 weeks = 126.9 μm ± 5.5 S.E.M, 4 weeks = 118.9 μm ± 6.1 S.E.M and 8 weeks = 116.5 μm ± 6.9 S.E.M (Fig. 2f). Based on CFP as well as OCT data we present a diagram showing what might occur in our laser-treated GA mouse model of AMD (Fig. 2g).

### Ultrastructural and inflammatory changes at the centre and margins of lesions correspond to early GA pathology

**Figure 3:**
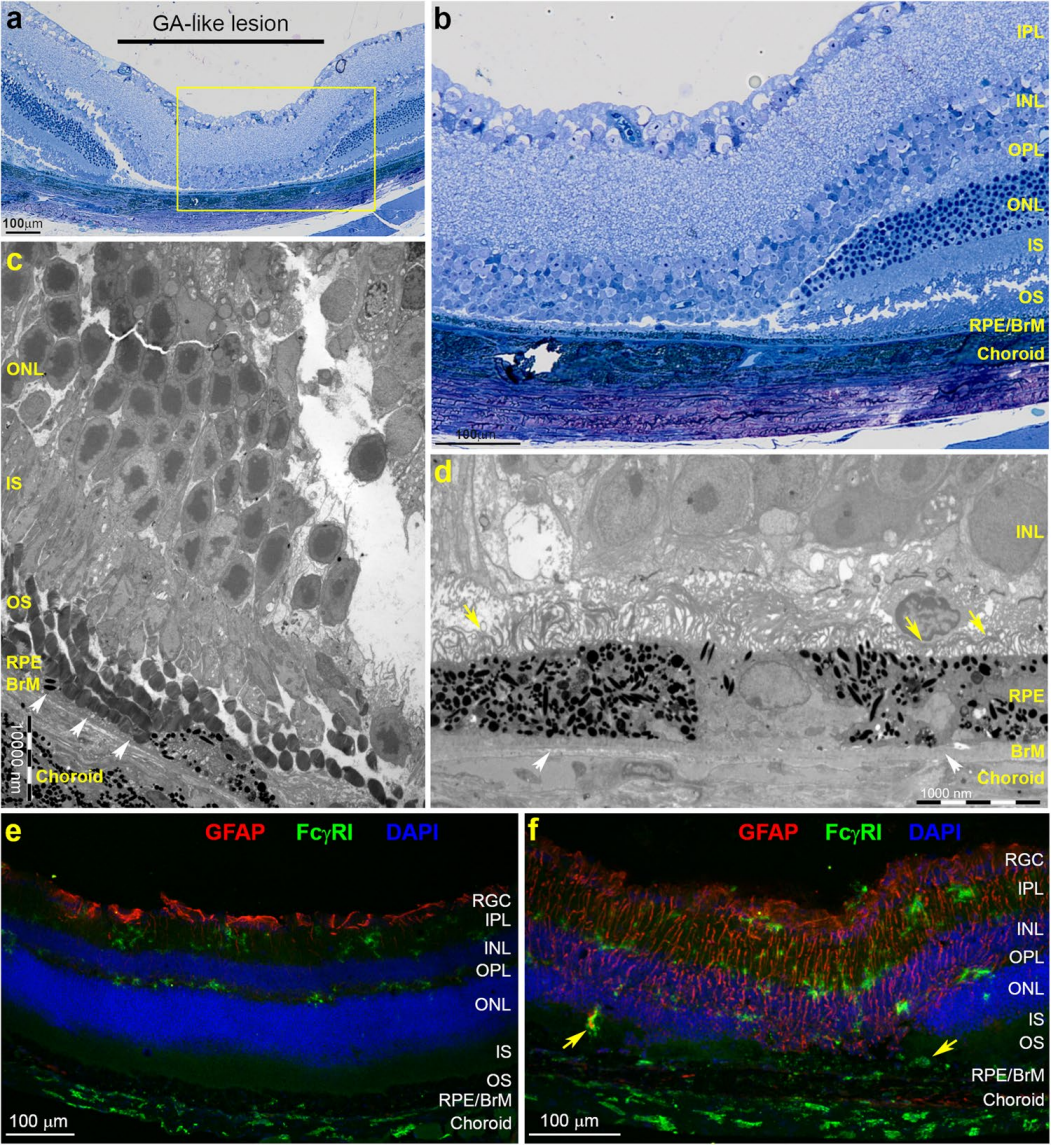
Structural changes and chronic inflammation within lesions and adjacent tissues in lasered mice correspond to early GA-like pathology. (**a**) Representative semi-thin tissue sections from an eleven month old mouse at 3 months post-treatment and stained with Toluidine blue show a characteristic GA-like lesion where photoreceptors are absent in lasered areas. (**b**) Magnified insert from (**a**) (yellow box) where the INL is observed lying in close apposition to the RPE. The inner retina had collapsed into the area that had been occupied by the degenerated photoreceptor layer. Lesion margins could be observed as well-defined wedge-shapes, with photoreceptor IS/OS and ONL as well as the OPL taking on a more normal appearance further away from the lesion. The RPE and BrM remains intact alongside a choroid that also appears to be unaffected. Scale bars in A and B corresponds to 100 μm. (**c**) Representative electron micrograph showing pathology in lesion margins. Areas where RPE cells had become atrophic can be observed (white arrows) alongside photoreceptor OS horizontally positioned next to the underlying BrM. However, the RPE monolayer was intact in most micrographs. Importantly, the BrM itself appears to remain unaffected. Photoreceptor OS followed by IS and the ONL gradually tapers closer to the lesion. (**d**) Micrograph from within the lesion showing the INL next to the RPE in the absence of a photoreceptor layer. The RPE monolayer shows neighbouring hypopigmented as well as hyperpigmented cells. Microvilli on the apical RPE surface appear to be disorganised and shorter in length (yellow arrows), whilst the BrM shows varying thicknesses (white arrows). Scale bars in c and d correspond to 1000 nm. (**e**) Representative confocal-immunofluorescence comparing GFAP (red) and FcγRI (green) staining in non-lasered tissues vs. (**f**) adjacent lasered areas at 4 weeks post treatment. Notice GFAP staining in processes extending into what appears to be remaining photoreceptors within lesions after 4 weeks, which had disappeared by 12 weeks when visualised by light and electron microscopy. GFAP expression was also observed in the INL and IPL layers, extending beyond the lesion into surrounding tissues. No GFAP expression was observed in non-lasered tissues except for constitutive staining near the vitreous interface. Upregulated FcγRI expression was observed clustered within lesions and in marginal tissues (yellow arrows). Only minimal FcγRI expression was observed in non-lasered tissues. Nuclei were labelled with DAPI and appear blue. Scale bars in e and f correspond to 100 μm. Retinal ganglion cells (RGC), Inner plexiform layer (IPL), Inner nuclear layer (INL), Outer plexiform layer (OPL), Outer nuclear layer (ONL), IS/OS (inner and outer segments) of photoreceptors and Retinal pigment epithelium (RPE).

Next, we assessed histological evidence of laser-induced retinal pathology in eyes from mice lasered 12 weeks previously. Toluidine blue straining of semi-thin sections provided evidence of an intact BrM and RPE monolayer within lasered areas, suggesting that treatment had not disrupted the BRB (Fig. 3a). Lasered regions were strikingly evident by a complete absence of the photoreceptor layer. Moreover, we found that tissues of the inner retina were displaced and now sat on the area created by the absent photoreceptors (Fig. 3a,b). Hence, the OPL was observed lying in close apposition to the RPE with some debris in between. Furthermore, we noted wedge-shaped tapering of the ONL and IS/OS layers at lesion margins (Fig. 3b, Supplementary Fig. S3). Tissues were also analysed by transmission electron microscopy (TEM), which confirmed loss of photoreceptors and wedge-shaped IS and ONL layers at lesion margins tapering into lasered-spots (Fig. 3c). Occasionally, we found evidence that some RPE in the monolayer were absent with OS lying directly apposed to BrM. However, in most lesions we observed an intact RPE, although these cells appeared to show pronounced abnormalities including hypopigmentation as well as hyperpigmentation; in some instances along neighbouring cells of the monolayer. We also observed disorganised and shortened microvilli on the apical surface where the INL had collapsed on to the underlying RPE (Fig. 3d). Debris between the RPE and the INL were discernible as photoreceptor outer segments (POS); which was all that remained of the obliterated photoreceptor layer (Supplementary Fig. S4). Of note, we found evidence of altered BrM within lesions, which were visible as abnormally thickened areas associated with highly invaginated basal RPE infolds (Supplementary Fig. S4). No pathological features were observed at the ultrastructural level in peripheral non-lasered areas or in control fellow eyes (Supplementary Fig. S4).

Cellular immunological changes within lesions of lasered mice were found as early as 4 weeks after laser treatment. Confocal-immunofluorescence revealed elevated glial fibrillary acidic protein (GFAP) in retinas of lasered mice (Fig. 3e,f), which appeared in the fragmented ONL within lasered spots. However, GFAP expression in the overlying INL and IPL extend beyond the immediate vicinity of lesions. The pattern of the microglial marker FcγRI also extends beyond the immediate locality of lesions. Images collated from locations distal to the laser injury site did not show any evidence of elevated inflammatory activity other than constitutive GFAP staining in the RGC layer, which stains for astrocytes rather than Müller cells (Fig. 3e,f). Corresponding brightfield images revealed clusters of pigmented cells in lasered spots that appeared to have separated from the underlying RPE (Supplementary Fig. S5). There was no evidence of any abnormal RPE in non-lasered regions. Four weeks after laser treatment, tissues were also assessed for evidence of VEGF upregulation or for any indication of choroidal neovascularisation in lasered sites compared to adjacent non-lasered regions (Supplementary S6). We found no evidence of elevated VEGF or nascent vessel formation at GA-like lesions.

### Early GA-like pathology corresponds to a functional impairment of the retina

**Figure 4:**
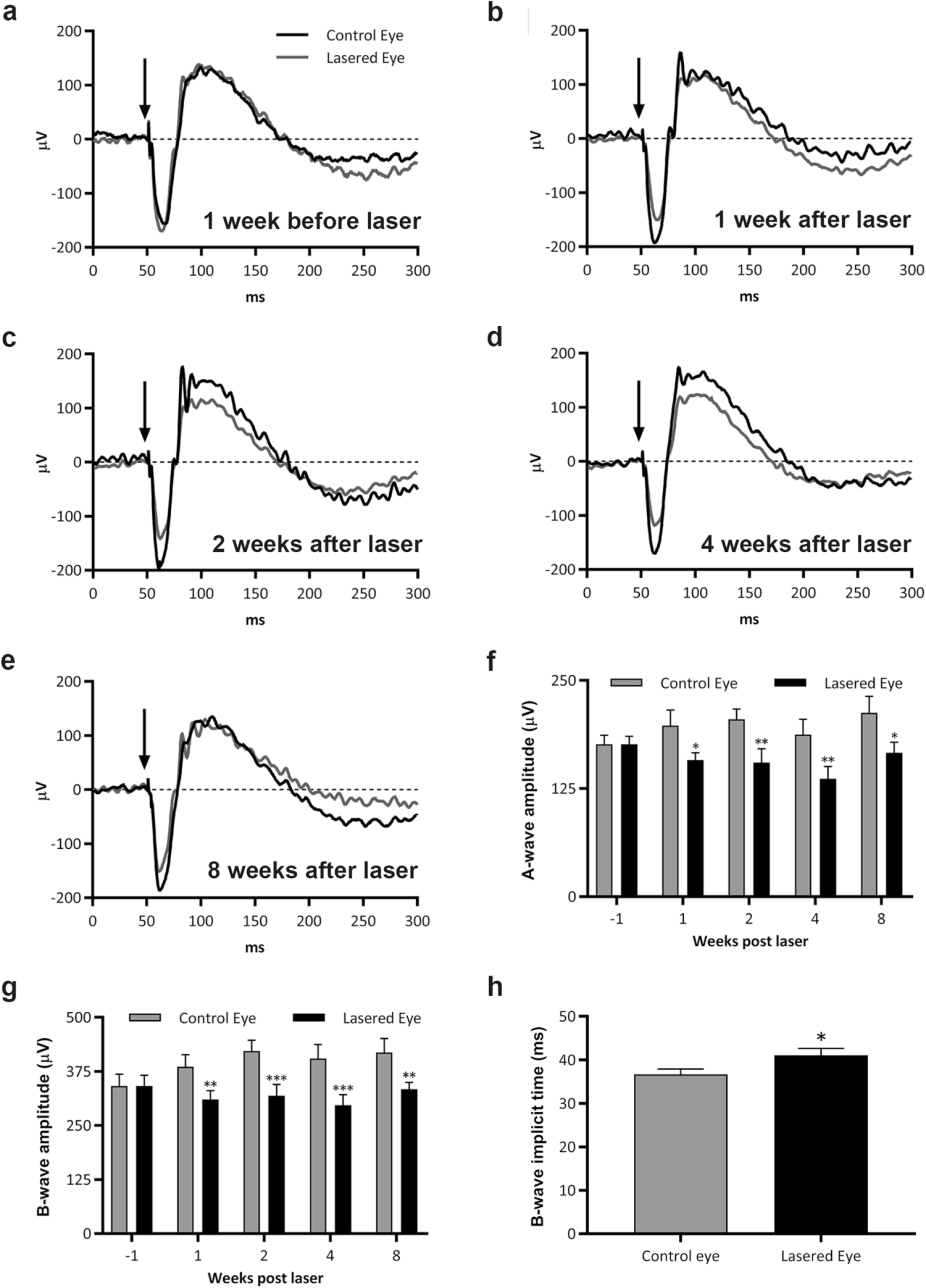
GA-like pathology corresponds to functional deficits in the mouse retina. Electroretinography (ERG) was used in longitudinal studies to measure the function of different retinal cell types. Average ERG traces are shown for lasered and non-lasered groups at (**a**) 1 week before laser, (**b**) 1 week after laser, (**c**) 2 weeks after laser, (**d**) 4 weeks after laser, and (**e**) at 8 weeks after laser. ERG values for lasered eyes were normalised to the fellow/control eye in each animal. (**f**) Comparison of A-wave amplitudes between lasered and non-lasered eyes. (**g**) Comparison of B-wave amplitudes, and (**h**) B-wave implicit times between lasered and non-lasered eyes. Arrows indicate point of the light stimulus. An average of 3 ERG measurements were obtained per eye at each time point (n = 8, five month old mice). Analysis by two-way ANOVA (repeated measures by both factors, factor 1: control vs. lasered eye, factor 2: time) followed with Holm-Sidak post-hoc testing. Graphs presented as mean ± SEM, significance denoted by *p < 0.05, **p < 0.01 and ***p < 0.0001.

Early AMD and GA patients show a reduction in A-wave (photoreceptor rods/cones) and B-wave (inner retina, predominantly Müller and ON-bipolar cells) amplitudes as well as increased B-wave implicit times during scotopic ERG assessments^16^. Consequently, we investigated these parameters in our model. Scotopic electroretinography (ERG) recordings set at the widest aperture to encompass the laser site and surrounding tissue were performed to non-invasively assess functional changes during progressive retinopathy. Post-hoc testing revealed a significant decrease in the A-wave amplitude of 20.2% (after 1 week), 24.4% (after 2 weeks), 27.1% (after 4 weeks) and 21.8% (after 8 weeks) post-laser (Fig. 4a–e,f). An overall A-wave reduction of 23.4% was detected between lasered and control eyes across all time points (F_1,7_ = 39.31, p = 0.0004). Post-hoc testing also revealed a significant reduction in the B-wave amplitude of 19.5% (after 1 week), 24.5% (after 2 weeks), 26.7% (after 4 weeks) and 20.4% (after 8 weeks) post-laser (Fig. 4a–e,g). We detected an overall deficit of 22.8% in B-wave amplitude between lasered and control eyes across all time points (F_1,7_ = 64, p ≤ 0.0001). There was also a significant difference in overall B-wave implicit times between lasered and control eyes (Fig. 4h) (n = 8 mice, with the non-lasered fellow eye acting as a control, F_1,7_ = 9.646, p = 0.0172). However, these did not reach significance between any time points in multiple comparison testing (Supplementary Fig. S7), although we observed a trend for increasing B-wave implicit times at 1, 2 and 4 weeks post laser.

### The transcriptional profile of laser-induced lesions correlates with genes upregulated in GA patients

**Figure 5:**
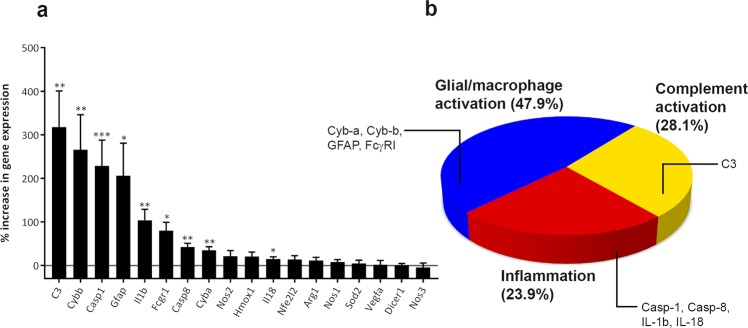
Genetic profiles of GA-like lesions in lasered mice correlate with genes upregulated in GA patients. (**a**) mRNA expression in retinas of mice at 4 weeks post laser measured by SYBR Green qPCR. Values are shown as an average increase in gene expression in lasered eyes compared to the non-lasered fellow eye of each animal. Pooled data for 11 animals (8 mice aged 5 months, and 3 mice aged 11–13 months). Multiple paired one-tailed t-test followed by Holm-Sidak multiple comparison correction. Significance denoted by *p < 0.05 and **p < 0.01. (**b**) A genetic profile of lasered retinas created by grouping upregulated mRNAs into distinct but potentially overlapping disease processes; (1) complement activation, (2) glial/macrophage activation and (3) inflammatory pathway.

Given the changes to immune cells in GA lesions, we also sought to determine the expression of a broad range of genes implicated in AMD-linked disease pathways. Based on persistent GA-like lesions, ERG deficits and chronic inflammation, we selected a 4 week time point following laser ablation for these studies. Comparison of mRNA expression between young adults (5 months) and aged mice (11–13 months) revealed no effect of age on GA lesions (Supplementary Fig. S8). Pooled data showed significant differences between lasered eyes compared with non-lasered eyes (Fig. 5a). Complement component C3 showed the highest upregulation (318%, *t*_10_ = 4.804, p = 0.0004). We also detected a 206% elevation of the astrocyte/Müller cell marker GFAP (*t*_10_ = 3.98, p = 0.0013) and the microglial marker FcγRI by 80% (*t*_9_ = 3.737, p = 0.0023) over non-lasered controls. In addition, genes associated with the inflammasome pathway were upregulated including Caspase-1 (228%, *t*_9_ = 8.423, p ≤ 0.0001), Caspase-8 (43%, *t*_10_ = 5.61, p = 0.0001), IL-1β (104%, *t*_10_ = 4.558, p = 0.0005) and IL-18 (15%, *t*_10_ = 3.271, p = 0.0042). We also detected elevated mRNA levels of NADPH oxidase pathway components p22phox (Cyb-a) and NOX2 (Cyb-b) by 35% (*t*_10_ = 4.426, p = 0.00064) and 266% (*t*_11_ = 4.381, p = 0.0007), respectively. In contrast, no significant changes were detected in the mRNA levels of nitric oxide synthesis enzymes Nos1 (*t*_11_ = 1.353, p = 0.103), Nos2 (*t*_10_ = 0.781, p = 0.2264) and Nos3 (*t*_10_ = 1.416, p = 0.1872). There were also no variations in the mRNA expression profiles of the oxidative stress-response genes Hmox1 (*t*_10_ = 1.963, p = 0.039), Nfe212 (*t*_10_ = 1.315, p = 0.1089) and Sod2 (*t*_10_ = 0.047, p = 0.817) or the wound-healing response gene Arg1 (*t*_10_ = 1.165, p = 0.1355). Importantly, we detected no changes in VEGF-A (*t*_10_ = 0.2591, p = 0.4004), or Dicer1 (*t*_10_ = 0.3019, p = 0.3845) (Fig. 5a). The mRNA changes in mouse GA-like retinas is summarised in a pie chart, where upregulated mRNAs were grouped into the following categories; (1) complement activation, (2) glial/macrophage activation and (3) inflammatory pathways (Fig. 5b).

## Discussion

Although events leading to GA are still incompletely understood, early stages include photoreceptor loss, which in most cases appear to precede patchy atrophy of the underlying RPE, BrM thickening and choroidal thinning^5–8^. This histopathology is observed alongside a remarkably intact inner retina. A longitudinal funduscopy study of nearly 100 GA patients revealed a defined sequence of events in nearly 65% of the individuals. (1) Drusen development (average > 6.6 years before GA onset), (2) Hyper-pigmented RPE (average > 5 years before GA onset), (3) Drusen regression and hypo-pigmented RPE (average > 2.5 years before GA onset) and (4) GA development^17^ that provides a roadmap of pathology to which with the exceptions of increased RPE autofluorescence and drusen formation, our mouse model can be compared. Here, we demonstrate how a 810 nm diode laser can be used to create a mouse model that develops early GA-like features. Our model has contrasting features with the laser-induced choroidal neovascularisation (CNV) mouse model described by Campochiaro and colleagues^18^, used widely to study nvAMD. Important differences include the type of the laser (810 nm diode laser vs. a krypton laser in the CNV model), its power and duration (32 mW for ~60 seconds vs. 350–400 mW for 0.05 seconds in the CNV model), and its diameter (400 μm vs. 50 μm in the CNV model). The laser wavelength does not appear to be critical to lesion development, as both beams operate above the absorption spectrum of xanthophyll pigment in the retina (420–500 nm) and haemoglobin in inner retinal blood vessels (450–550 nm), hence penetrate the neuroretina without causing apparent damage. The laser is instead absorbed by melanin (400–1000 nm), which is expressed in the RPE/BrM/choroid complex^19^. Therefore, by adjusting the laser power and duration a GA-like lesion can be induced. Critically, there was no evidence that laser exposure had induced BrM breaks as evaluated by FA or at ultrastructural level, which differentiates our model with laser-induced CNV^18^. There was also no evidence of VEGF mRNA or protein upregulation in GA-like lesions after 4 weeks. By contrast, VEGF transcripts were upregulated in the CNV model soon after laser treatment^20–22^, with VEGF proteins elevated for up to 4 weeks^23^.

Using CFP, we observed GA-like lesions with well-defined borders in lasered mice as early as 1 week after treatment. Over the following weeks, we noticed lesions becoming more hyperpigmented whilst their margins became more defined and less hazy. GA lesions of patients also contain discernible margins, although we are unaware of any reports suggesting that fluorescence in these borders change over time. Nonetheless, the assessment of autofluorescence patterns and lesion size as predictors of evolving pathology is an important aspect of clinical evaluation^24^. In GA patients, areas of hyper-reflectivity in OCT scans are associated with the presence of drusen, calcified or regressed drusen, inflammation, fibrosis, and/or cell death^25^. OCT scan of our lasered mice revealed how pathology affects distinct retinal layers, although hyper-reflectivity due to drusen or related changes can be excluded in our model. We observed the absence of the ONL and IS/OS with some effects on the OPL as well as the RPE-BrM; a pattern of damage also observed in retinas of GA patients^8,25^. At margins of lesions, IS/OS reflectivity is lost whilst retaining the underlying RPE. ERGs of GA patients reveal such photoreceptors at lesion margins retain their function even though the IS/OS are not evident^25^. Analysis of retinal thickness revealed a successive reduction until 4 weeks, after which there were no further changes. Reduced retinal thicknesses in GA patients have been well documented^7,8^, although they do not appear to be necessarily correlated with diminished visual acuity^26^.

Histological analysis revealed characteristic GA features including hyperplastic RPE and those with disorganised/shortened microvilli. We also observed shedding of pigmented atrophic/RPE cells into the subretinal space and membranous debris between the collapsed INL and RPE, which were recognisable as leftover OS, presumably after the death of photoreceptors. BrM thickening was associated with highly invaginated basolateral RPE membranes. However, the most striking feature was the complete absence of photoreceptors in lasered areas, relative to adjacent/non-lasered tissues, and association of the inner retina with RPE/BrM. Light and electron microscopy also revealed a gradually diminishing radius of damage from the lesion that bore striking resemblances to the junctional zone and areas of severe/moderate degeneration in patients^7^. These can extend up to 1400 μm from the GA edge^8^ and were observed at a reduced scale in our model. Immediately adjacent to the lesion, characteristic wedge-shaped GA-like margins were evident, where the outer retinal layers were tapered at margins of the laser site. Additional early GA pathology in lesion margins described by Sarks and colleagues and observed in these mice included atrophic photoreceptors in the presence of an intact/underlying RPE. Atrophic photoreceptors in the periphery of lasered areas appear to be similar to findings showing that only a minority of GA patients retain appreciable photoreceptors at lesion margins^8^. Confocal-immunofluorescence studies of lasered eyes added to this picture of graded damage surrounding lesions. GFAP is expressed by astrocytes under resting and active conditions, and by Müller cells under active conditions^27^. We observed GFAP expression in deeper retinal layers, focused within lesions containing remaining photoreceptors. However, GFAP staining was also observed in the overlying INL and IPL, extending to margins of the lesion but absent in distal non-lasered tissues, suggesting that Müller cells were predominantly activated in lesions and in immediate tissues. While GFAP expression alone cannot distinguish between activated astrocytes and Müller cells, the staining pattern away from the vitreous interface appears typical of the Müller cell processes that span the retinal layers. Activated Müller cells reported in donor AMD tissues^28^ therefore also appears to be recapitulated in our model. Astrogliosis has also been described in early AMD patients^28^. However, due to strong constitutive expression of astrocytic GFAP at the vitreous interface we were unable to determine whether this was the case in our model. FcγRI expressed constitutively on myeloid cells were also detected within lasered spots, but only minimally in distal healthy tissues. Activated myeloid cells are frequently observed in GA patients^29^ and may represent resident retinal microglia or infiltrating macrophages. Our previous work described a mouse model of retinopathy where microglial activation and recruitment of CD45^+^ cells from the periphery were dependent on presence of the γ-chain used by FcγRI^14^. Myeloid cells are recruited to the RPE-BrM in increasing numbers as early AMD progresses to intermediate stages, but then decline in GA as tissues become atrophic, although those few remaining cells appear highly activated^29,30^. Microglia may be recruited to the lesioned RPE-BrM/choroid complex in our GA-like model to engulf melanin/autofluorescent-laden cells, which we observed being shed into the subretinal space. However, we cannot speculate on the type/origins of microglia/macrophages without further studies. Comparison of VEGF protein levels and the basement membrane marker laminin between lasered and adjacent non-lasered areas showed no evidence of angiogenesis in this GA-like model.

Photopic and scotopic multifocal ERGs assessments in GA patients show an almost complete loss of cone/rod responses within lesions. Cone-mediated responses in patients also diminish at GA borders, returning to normal levels beyond lesion margins^25^. Scotopic ERG responses in GA patients, which measure retinal function under low-light conditions, are more sensitive at revealing functional changes in areas whereas photopic multifocal recordings appear normal^25^. Our scotopic ERG recordings revealed that deficiencies in A and B-waves were remarkably similar in the mouse model, indicating that photoreceptor deficits also resulted in reduced signalling to Müller/ON-bipolar cells. These findings were noteworthy given that focal ERGs were recorded at the largest aperture setting and therefore encompassed the lesion, its marginal zone as well as some degree of surrounding healthy tissue. Diminished ERG recordings were also broadly similar between 1 week and 8 weeks following treatment, indicating no further functional decline in inner and outer retinal layers. ERG deficits in GA patients are remarkably consistent over many years^3^, a feature that appears to be recapitulated in our model. Prolonged implicit times of scotopic A and B-waves were also reported in GA patients, which could underlie pan-retinal pathology including inflammation or ischemia, prior to disease onset^31^. Reduced B-wave implicit times was also observed in lasered eyes compared to non-lasered controls but without significant differences between successive weeks.

The mRNA profile of inflammatory genes implicated in GA^32–34^ was studied in our model and grouped into (1) the complement/complement regulatory pathway, (2) glial/macrophage activation and (3) inflammatory pathways. The largest upregulation was observed in complement C3, which has a key pathogenic role in the senescent retina. Mutations/variants of complement proteins and their regulators accounts for ~50% of AMD cases, with the highest associations per locus found in the C3 gene^33^. C3 and its breakdown products have been reported in the neuroretina, in drusen, BrM as well as the choroid^35^. Amongst the glial/macrophage activation pathway, we detected a substantial upregulation of GFAP mirroring confocal-immunofluorescence data. Others upregulated in this group includes FcγRI which we also observed by confocal-immunofluorescence, as well as Cyb-a and Cyb-b which are components of the NOX (NAPDH oxidase) phagocytosis pathway^36^. The expression of anti-oxidant genes Hmox-1, Nfe212 and Sod2 genes however were unaltered, reflecting a balance towards oxidative stress although these may be activated earlier. The NOX pathway has also been implicated in other neurodegenerative conditions such as Alzheimer’s disease^37^. mRNA upregulated in the inflammatory pathway included Casp-1, Casp-8, IL-1β and IL-18. Activated caspase-1 (part of the NLRP3 inflammasome), can induce cell death by pyroptosis and/or cleave pro-inflammatory cytokines into mature bioactive forms including IL-1β and IL-18^38^. Caspase-1 involvement has been widely reported, including activated caspase-1 and NLRP3 in macrophages surrounding drusen-like deposits in a dry AMD mouse model^39^. Caspase-8 was also upregulated in RPE cells from donor GA retinas^40^. Treatment of cultured RPE by *Alu* RNA activated caspase-8, whose effects are thought to act further downstream of inflammasome activation. Caspase-8 can also activate caspase-1 and is involved in inflammasome-mediated IL-1β processing. Recent findings described a pathway through which *Alu* RNA accumulation and IL-18 upregulation led to death of RPE cells via activation of capase-8 through a Fas ligand-dependent mechanism^40^. Dicer1 levels were decreased in GA patients leading to *Alu* RNA accumulation and inflammasome activation^41^. However, Dicer 1 was unaltered, indicating that inflammasome activation was likely through an alternative mechanism due to the acute nature of our model. We also saw no evidence of VEGF mRNA upregulation, a potent activator of angiogenesis and neovascularisation^3^, suggesting that our laser-induced mouse model favours a GA phenotype. The RNA profile of lasered mouse retinas thus recapitulates the genetic make-up of GA retinas to a remarkable extent.

**Figure 6:**
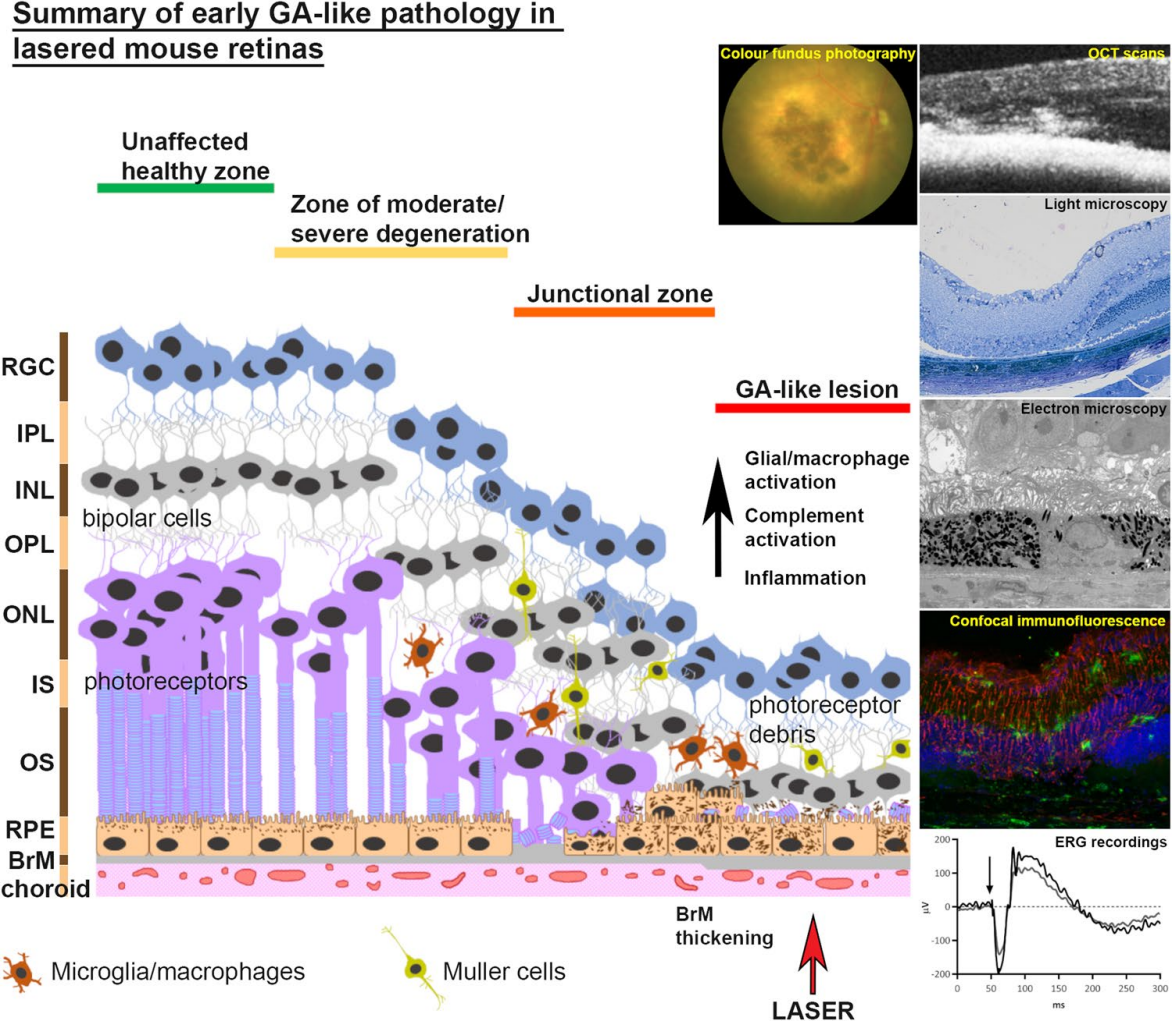
A diagrammatic figure summarising early GA-like pathology in lasered mouse retinas. Based on colour fundus photography, OCT, light and electron microscopy, confocal-immunofluorescence, ERG as well as genetic profiling, we present a summary of histopathological features in a representative lasered mouse retina at 12 weeks post treatment. Distinct zones of GA-like histopathology can be identified in a manner akin to those described by Sarks and colleagues in donor GA tissues. Structural changes are associated with functional retinal defects as well as activation of glial/macrophage, complement and inflammatory pathways reported in GA patients. With rapid disease onset, a high degree of experimental reproducibility as well as other important disease features, this model is useful for studying early stages of GA.

Here, we report the first development and characterisation study of a novel laser induced early GA-like mouse model of retinal degeneration or the “Southampton AMD model”. This model possess many of the important early GA features, which are summarised in a diagram (Fig. 6). The persistence of GA-like features as long as 3 months after laser treatment is noteworthy, and contrasts to neovascular features in the CNV model that manifest soon after lasering. Its acute nature however limits scope for studies into lipofuscin accumulation, a major index of RPE aging, as well as drusen pathophysiology^42,43^. These limitations notwithstanding, the importance of developing better GA-like models has also been demonstrated recently using brown Norway rats^44^. Our model may be useful to study early GA features including RPE abnormalities, BrM thickening and neuroinflammation, assess functional retinal defects and genetic susceptibility as well as cholesterol dysregulation^5–8,34^. It could also be used to evaluate immunotherapies currently tested in transgenic mice^45–48^ as the ability to rapidly generate reproducible early GA-like pathology in a matter of weeks and in a cost-effective manner are particularly attractive features. Investigators could incorporate AMD risk factors such as ageing and unhealthy diet to further exploit the advantages offered by this model. Future studies will evaluate whether initial pathogenic RPE changes in lesions will eventually progress to outright RPE atrophy or indeed include wider damage to photoreceptors which exceeds the area of RPE loss. This has the potential to add further value to this surrogate model of early GA-like pathology, as it could be used to study terminal GA in living eyes for which a fully satisfactory animal model still does not exist.

## Methods

### Animal experiments

All experiments were performed on female C57BL/6J mice (Charles River, UK) and bred at the University of Southampton, Biomedical Research Facility. Mice had water and standard Chow diet (SDS, UK) *ad libitum* and were maintained in a standard 12 hour dark/12 hour light cycle. 94 mice between the ages of 5–13 months were used in the development of this model. Animal husbandry and procedures were performed within the guidelines of the University of Southampton Research Ethics Committee and under a UK Home Office licence. All procedures were conducted in accordance with the ARVO (Association for Research in Vision and Ophthalmology) statement for the use of animals in ophthalmic and vision research. Experimental protocols were approved by the Universities’ Animal Welfare and Ethical Review Body (AWERB). All drugs required for laser treatment and *in vivo* assessment of the retina were supplied by Centaur Services, UK. Mice were given reversible anaesthesia comprised of 6% ketamine, 10% dexdomitor in sterile saline at 10 μl/g of mouse weight. Anaesthesia was reversed at the end of the procedure with 10% antisedan in sterile saline at 10 μl/g of mouse weight. Mouse eyes were maximally dilated using 2.5% phenylephrine eye drops for >1 minute followed by 1% tropicamide eye drops for >1 minute. For terminal procedures, mice were anaesthetised with rat avertin (3% w/v 2,2,2′-tribromoethanol, 7.2% ethanol, 1.8% tertiary amyl alcohol in 0.9% NaCl from Sigma-Aldrich, UK). While anaesthetised with avertin, mice were trans-cardially perfused with 0.9% NaCl containing 5 units/ml heparin (CP Pharmaceuticals, UK) before eyes were removed for studies with the exception of ultrastructural analysis, where eyes were collected following cervical dislocation.

### Induction of GA-like lesions by laser treatment

Atrophic retinal lesions were induced using a fibre-coupled 810 nm continuous wave diode laser (CNI optoelectronics Tech Co, China) and laser injector attachment on a Micron III retinal imaging system (Phoenix Labs, CA, USA). The 810 nm laser has a near infrared wavelength enabling deeper penetration of retinal layers and is mainly absorbed by the RPE-choroid^19^. The laser injector has a camera for obtaining CFP, which enables orientation and visualisation of the lasered area. Mice were orientated on a stage so that the optic nerve was on the right hand side in each image, following which the retina was targeted with a focused laser beam (400 μm diameter) at the following settings: low-power (22 mW), medium-power (32 mW) and high-power (42 mW). Laser treatment was performed until a faint subthreshold burn, a very faint retinal whitening or just discernible whitening was apparent (average 60 seconds per spot). Mice received multiple laser spots (average 7) until the field of view was lesioned, except near the optic nerve, which was spared. The areas were lasered in close apposition to enable lesions to develop confluence.

### *In vivo* imaging

Fluorescein angiography (FA) was recorded using a Micron III camera. For FA, mice were injected i.p with 100 μl of 2.5% w/v fluorescein (Bausch and Lomb, UK) in physiological saline. Animals were orientated on a stage so that the optic nerve would be visible in the same location in each image and images acquired with a 490 nm light filter at 5, 10 and 15 minutes post injection using Phoenix Micron III retinal imaging microscope software (Phoenix Labs, CA, USA).

OCT scans were obtained using a Bioptigen Envisu R machine (Leica Microsystems, IL, USA). 0.2% carbomer liquid eye gel was replaced with Systane Lubricant Eye Drops (Alcon, UK). Mice were orientated on a stage in the manner described for acquiring CFP to obtain images in the same orientation. 100 B-scans (each an average of 25 individual B scans comprising 1000A scans) were captured using InVivoVue v2.3 (Leica Microsystems, IL, USA) to provide an image of the retinal layers over a 1.4 mm^2^ area of retina. ImageJ software (NIH, USA) was used to track average retinal thickness by manually demarcating the area between the retinal ganglion cells (RGCs) and choroid across a 560 μm length of the retina encompassing the lesion site. Three scans across the lesion were averaged at each time point, and similar scan locations were taken amongst time points to reduce variability.

Scotopic ERGs were obtained using a Micron III retinal imaging system with a focal ERG attachment (Phoenix Labs, CA, USA). Mice were dark-adapted overnight and the procedure carried out under low-level red light. The corneal electrode (gold) was attached to the focal ERG lens mount, whilst the reference and ground electrodes (platinum) were inserted under the skin between the ears and adjacent to the tail respectively. Mice were exposed to three flashes of white light at 6.8 log cd.s/m^2^ intensity for 1 ms, with each flash separated by 120 s to restore dark adaptation. Recordings were obtained for 50 ms pre and 250 ms post stimulus using LabScribe ERG v3 software (iWorx, Dover, NH, USA) with a 2–1000 Hz band pass filter, 5 kHZ sampling frequency and 50 Hz noise reduction. An average of 3 readings were obtained per eye at each time point. A-wave and B-wave amplitudes as well as B-wave implicit time for each retina was calculated to assess photoreceptor and inner retinal function. The ERG value for the lasered eye was normalised to the control/non-lasered fellow eye for every mouse in each session. This approach helped to limit potential variations in ERG recordings between different sessions.

### Light and transmission electron microscopy

The anterior pole of enucleated eyes were removed and posterior eye cups immersed in a primary fixative comprising 3% glutaraldehyde in 0.1 M cacodylate buffer and 2 mM CaCl_2_ at pH 7.2. The specimens were then rinsed in 0.1 M cacodylate buffer, 0.23 M sucrose and 2 mM CaCl_2_ at pH 7.2, post-fixed in 2% osmium tetroxide in 0.1 M cacodylate buffer for 2 hours, rinsed in buffer, and the block stained with 2% aqueous uranyl acetate. The specimens were dehydrated and embedded in Spurr resin (Agar Scientific, UK). 0.5 μm resin semi-thin sections were cut on a Reichert Ultra cut E ultramicrotome (Leica Microsystems, IL, USA), stained with 1% Toluidine blue and 1% Borax, and viewed under a light microscope. Gold ultrathin sections were cut on the ultramicrotome and stained with Reynolds lead, and viewed on a Hitachi H7000 (Hitachi High Technology, Japan) transmission electron microscope fitted with a SIS Megaview III plate camera (EMSIS, Germany).

### Immunohistochemistry

Enucleated eyes were prepared for immunohistochemistry by embedding eyes in optimal cutting temperature medium (VWR, UK) and rapid freezing with isopentane on dry ice following trans-cardial perfusion. Eyes were stored at −20 °C until use. Immunohistochemistry experiments were carried out in 20 μm cryo-sections of fresh-frozen eyes as described previously^14^. Sections were stained with primary antibodies against rat anti-mouse FcγRI (Clone 152-9, a kind gift from Dr Alison Tutt, University of Southampton), rabbit anti-human GFAP (Dako, Denmark), laminin (L9393, Sigma, UK) and VEGF (MA5-12184, Invitrogen). Secondary antibodies used were donkey anti-rat IgG-AF488, goat anti-rabbit IgG-AF568 (ThermoFisher, UK), goat anti-mouse IgG1 AF488, goat anti-rabbit IgG-AF568 (A11011, Invitrogen) and 4′6′-diamino-2-phenylindole (DAPI) for nuclei staining. Z-stack images acquired using a Leica SP8 (Leica Microsystems, IL, USA) confocal laser scanning microscope were converted into a maximum intensity projection in ImageJ (NIH, USA). Fluorescence was quantified in the whole field of view for separate channels using the histogram analysis function in ImageJ (NIH, USA) and shown as mean, modal, minimum and maximum values.

### mRNA analysis

Retinas were isolated following trans-cardial perfusion, snap-frozen in liquid nitrogen and stored at −80 °C until use. For mRNA analysis, individual retinas were homogenised in 800 μl TRIzol reagent (ThermoFisher, UK) using motor-driven pellet pestles. After 1 hour incubation, 80 μl of 1-bromo-3-chloropropane (Sigma-Aldrich, UK) was added, the mixture shaken vigorously for 15 seconds and incubated for 3 minutes followed by centrifugation at 12,000 g for 15 minutes at 4 °C. 300 μl of resultant RNA-containing aqueous phase was purified using the PureLink RNA Mini Kit (ThermoFisher, UK) according to the manufacturers’ instructions. 250 ng RNA was transcribed into cDNA using the TaqMan Reverse Transcription Reagents kit (ThermoFisher, UK) following the manufacturers’ instructions for cDNA synthesis with random hexamers. qPCR analysis was performed in duplicate using 1x iTaq Universal SYBR Green Supermix (Bio-Rad, UK) with primers (Supplementary Tables S1–S3). The efficiency of each primer pair was calculated based on five serial dilutions (5-fold) of retinal RNA. Relative RNA levels were calculated using the Pfaffl method^49^ to take into account differences in primer efficiency compared to the housekeeping gene GAPDH.

### Statistical analysis

GraphPad Prism v7 (CA, USA) was used to perform statistical analyses and create graphs of mean ± SEM for each experimental group. All data passed tests for normality and similar variances between groups using Shapiro-Wilk and Bartlett’s test respectively. For mRNA analysis, multiple parametric one-tailed t-tests (paired for control vs lasered eye) were performed for each gene followed by Holm-Šídák multiple comparisons correction. For ERG analysis, amplitude and implicit time for A-waves and B-waves were calculated and analysed by two-way ANOVA (repeated measures by both factors, factor 1: control vs. lasered eye; factor 2: time) followed by Holm-Šídák post-hoc testing. For OCT thickness quantification, average retinal thickness values across the lesion site were calculated and one-way ANOVA (repeated measures: control vs lasered eye) was performed followed by Holm-Šídák post-hoc testing.

## Supplementary information

Supplementary Information

## Data Availability

Data generated during this study are available from the corresponding author on reasonable request.
