# Supplementary material for: A lasered mouse model of retinal degeneration displays progressive outer retinal pathology providing insights into early geographic atrophy
Source: Sci Rep. 2019 May 16;9:7475. doi: 10.1038/s41598-019-43906-z (PMC6522499; doi:10.1038/s41598-019-43906-z)
Supplement: Supplementary file 1 — Supplementary Information [file 41598_2019_43906_MOESM1_ESM.pdf]

Title: A lasered mouse model of retinal degeneration displays progressive outer retinal pathology providing insights into early geographic atrophy.

Authors: Paul Ibbett[a,†], Srinivas V. Goverdhan[b,c,†], Elena Pipi[a], Joe K. Chouhan[a], Eloise Keeling[b], Elizabeth M. Angus[d], Jenny A. Scott[b], Maureen Gatherer[b], Anton Page[d], Jessica L. Teeling[a], Andrew J. Lotery[b,c,*], J. Arjuna Ratnayaka[b,*].

Affiliations:

a: Biological Sciences, University of Southampton, SGH, South Lab and Path Block, MP840, Tremona Road, Southampton, SO16 6YD, United Kingdom.

b: Clinical and Experimental Sciences, Faculty of Medicine, University of Southampton, MP806, Tremona Road, Southampton SO16 6YD, United Kingdom.

c: Eye Unit, University Hospital Southampton NHS Foundation Trust, Southampton, SO16 6YD, United Kingdom.

d: Biomedical Imaging Unit, University of Southampton, MP12, Tremona Road, Southampton SO16 6YD, United Kingdom.

[†] Contributed equally to this work (joint first authors)

***** Corresponding authors: J. Arjuna Ratnayaka and Andrew J. Lotery.

J. Arjuna Ratnayaka: Clinical and Experimental Sciences, Faculty of Medicine, University of Southampton, MP806, Tremona Road, Southampton SO16 6YD, United Kingdom. Tel: +044 238120 8183, Email: J.Ratnayaka@soton.ac.uk

Andrew J. Lotery: Clinical and Experimental Sciences, Faculty of Medicine, University of Southampton, MP806, Tremona Road, Southampton SO16 6YD, United Kingdom. Tel: +044 238120 5049, Email: A.J.Lotery@soton.ac.uk

Supplementary information (figures and tables)

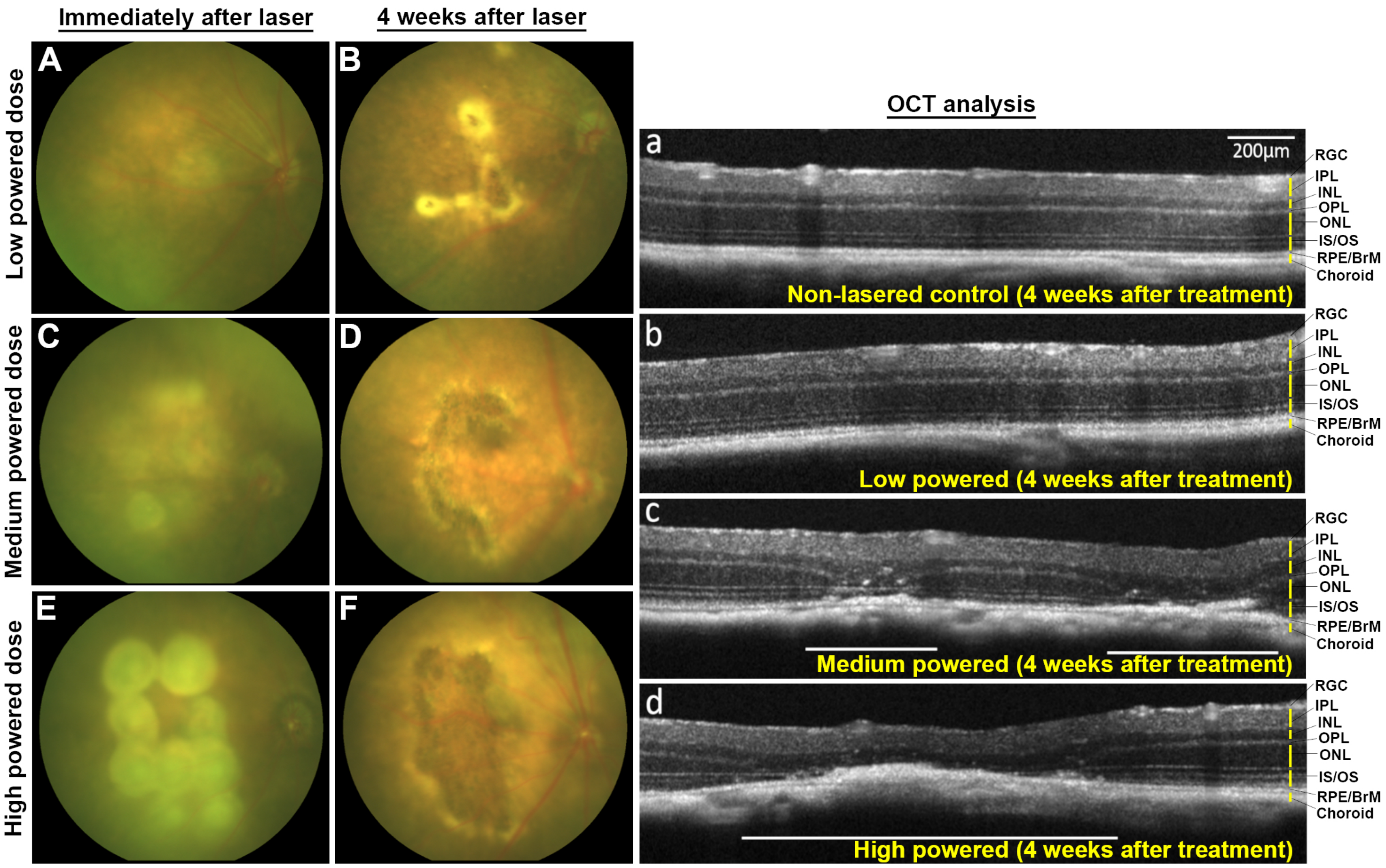

Figure S1: Optimisation of laser treatment. Multiple and adjacent areas of each retina were targeted with either [A,B,b] a low-powered (22mW, 60s), [C,D,c] medium-powered (32mW, 60s) or [E,F,d] a high-powered (42mW, 60s) laser (n=6 mice/group, 5 month old mice). Development of lesions were assessed by funduscopy and longitudinal OCT. [A-F] Representitive funduscopy images showing formation of lesions immediately after laser treatment and following 4 weeks. Treatment with a low-powered laser failed to cause atrophic lesions after 4 weeks with any reproducibility, except on rare occassions. By contrast, a medium-powered laser resulted in consistently generating lesions after 4 weeks. A high powered-laser caused the rapid development of a confluent lesion, but also damage to the overlying neuroretina. Discernable areas of the lesions in OCT images are indicated by a white line.

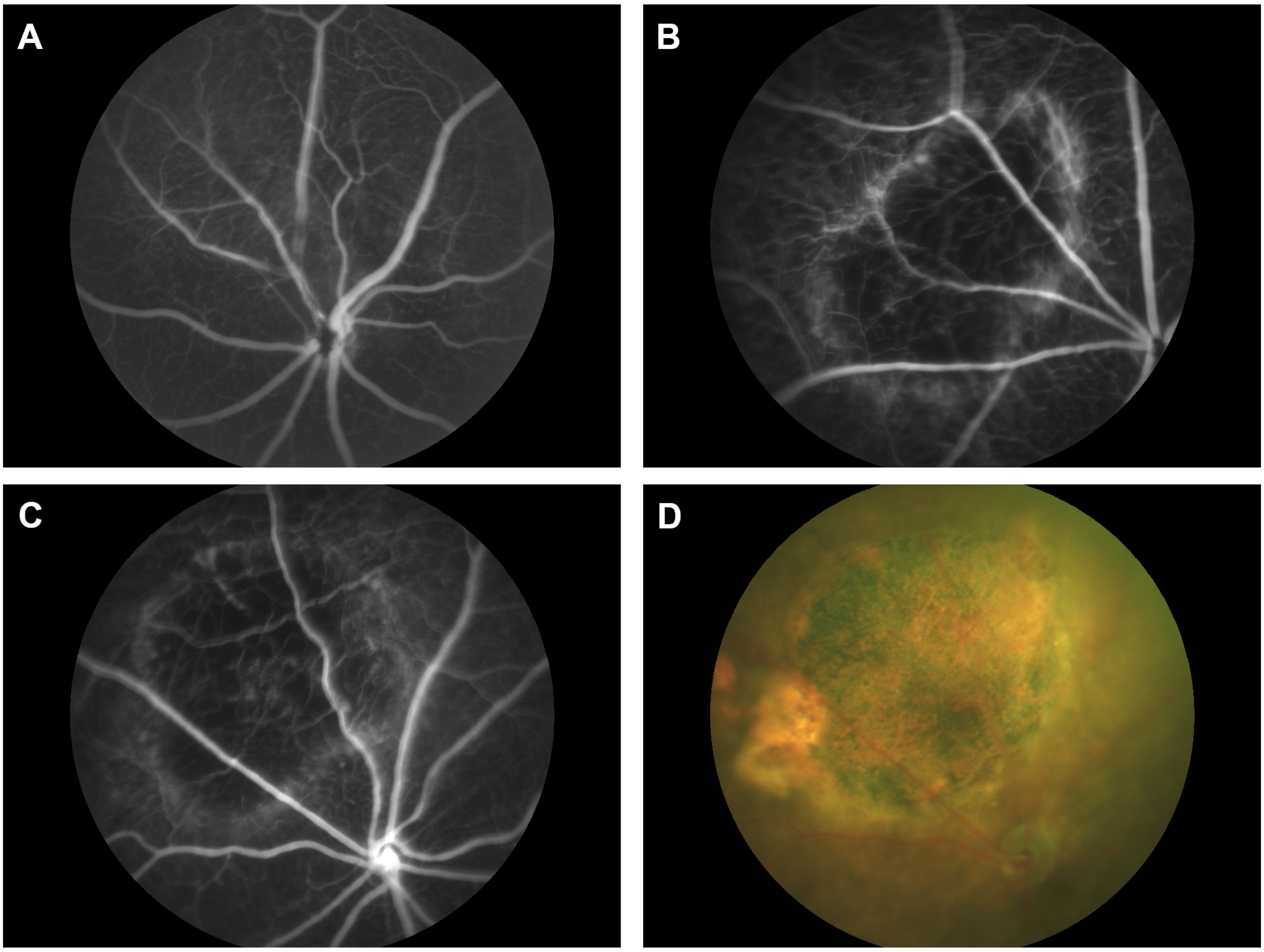

Figure S2: Fluorescein angiography (FA) was performed to assess potential breaks in the blood-retinal -barrier. Representitive FA performed in 13 month old mice showing [A] a healthy non-lasered retina and [B] a retina 8 weeks after laser treatment. [C] Lasered eye and [D] colour fundus photograph (CFP) showing lesion with well-defined borders and visible choroidal vessels. Notice the hyperfluorescent marginal zone of lesions. There was no evidence of dye leakage.

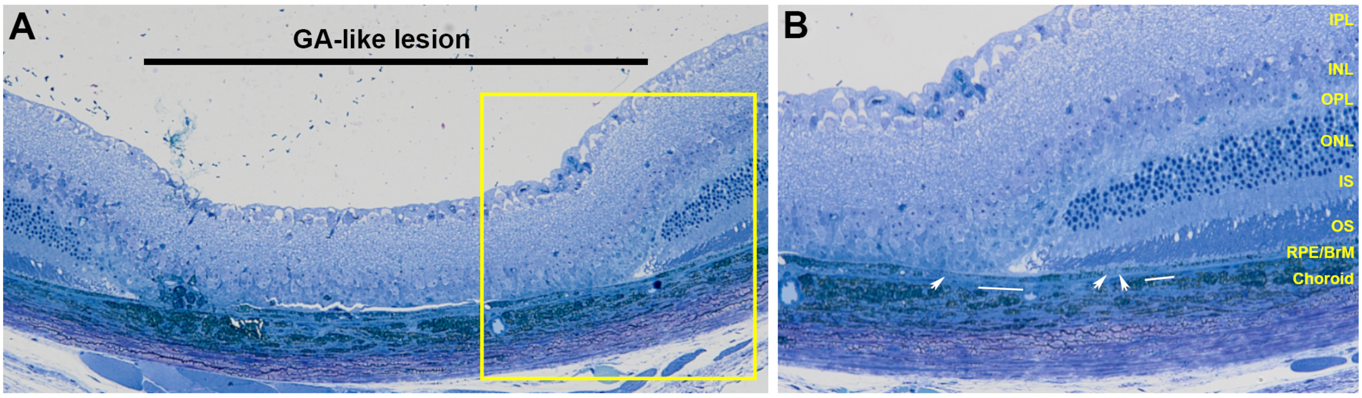

Figure S3: Semi-thin light microscopy sections showing GA-like lesions. [A] Mouse eyes 3 months after laser treatment shows a characteristic lesion where the photoreceptor layer had been obliterated. [B] Inset shows the RPE monolayer to be intact, although some areas of thining (white bars) was evident. Areas of hypo-pigmented RPE (white arrows) can also be observed.

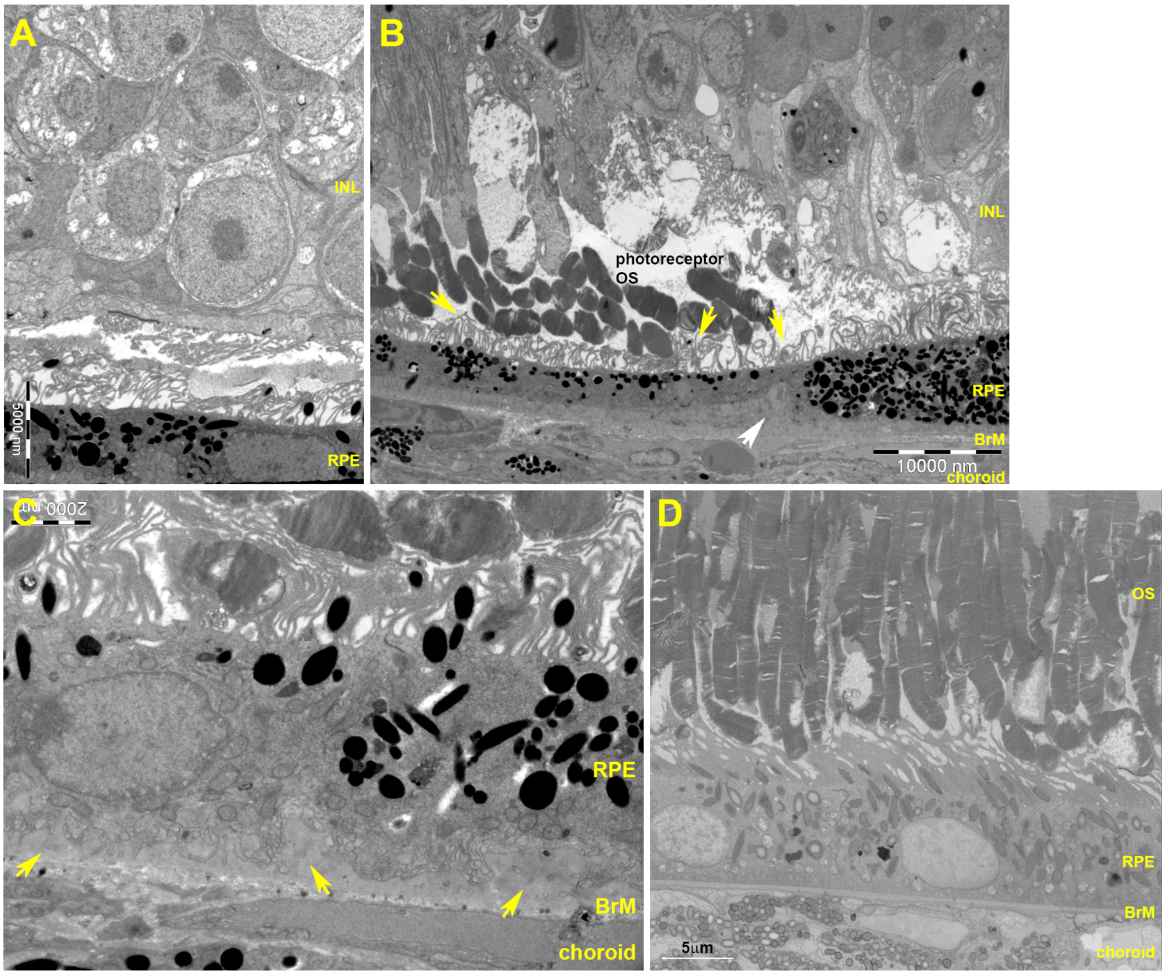

Figure S4: Ultrastructural abnormalities within GA-like lesions in tissues of the outer retina. Mouse eyes were assessed by TEM three months after laser treatment. [A] Representitive electron micrograph showing an intact RPEmonolayer on to which the INL had collapsed in the absence of photoreceptors within lasered spots. Disorganised microvilli on the apical RPE surface were also visible amongst debris. [B] Micrograph showing debris within lesions, some of which can be recognised as POS overlying the RPE. Areas where microvilli had become shortened and disorganised are indicated by yellow arrows. The RPE monolayer was intact although hypo and hyper-pigmented cells were evident. In some areas, we also observed an unusual thickening of BrM (white arrow). [C] A high-powered electron micrograph showing highly invaginated basal infolds of the RPE corresponding to thickened BrM (yellow arrows) within lasered spots. [D] Non-lasered mouse eye showing normal histology of the outer retina.

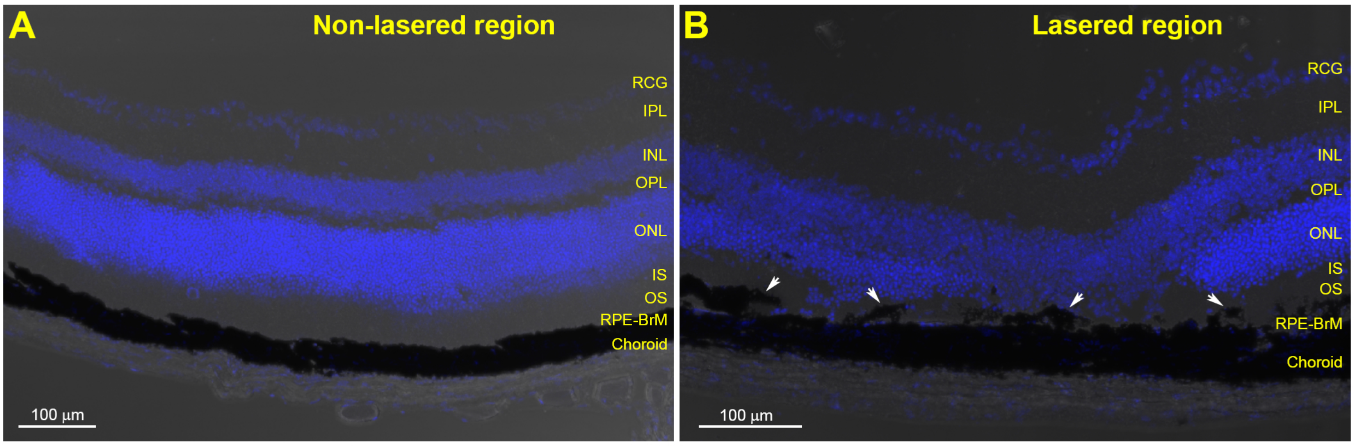

Figure S5: Chronic inflammation in lesions are associated with abnormal RPE. Corresponding brightfiled images of confocal immunofluorescence panels in figure 3E and 3F. [A] Non-lasered vs. [B] lasered spot showing evidence of abnormal/hyperplastic RPE (white arrows in B). DAPI nuclear staining is shown in blue. Scale bar corresponds to 100µm.

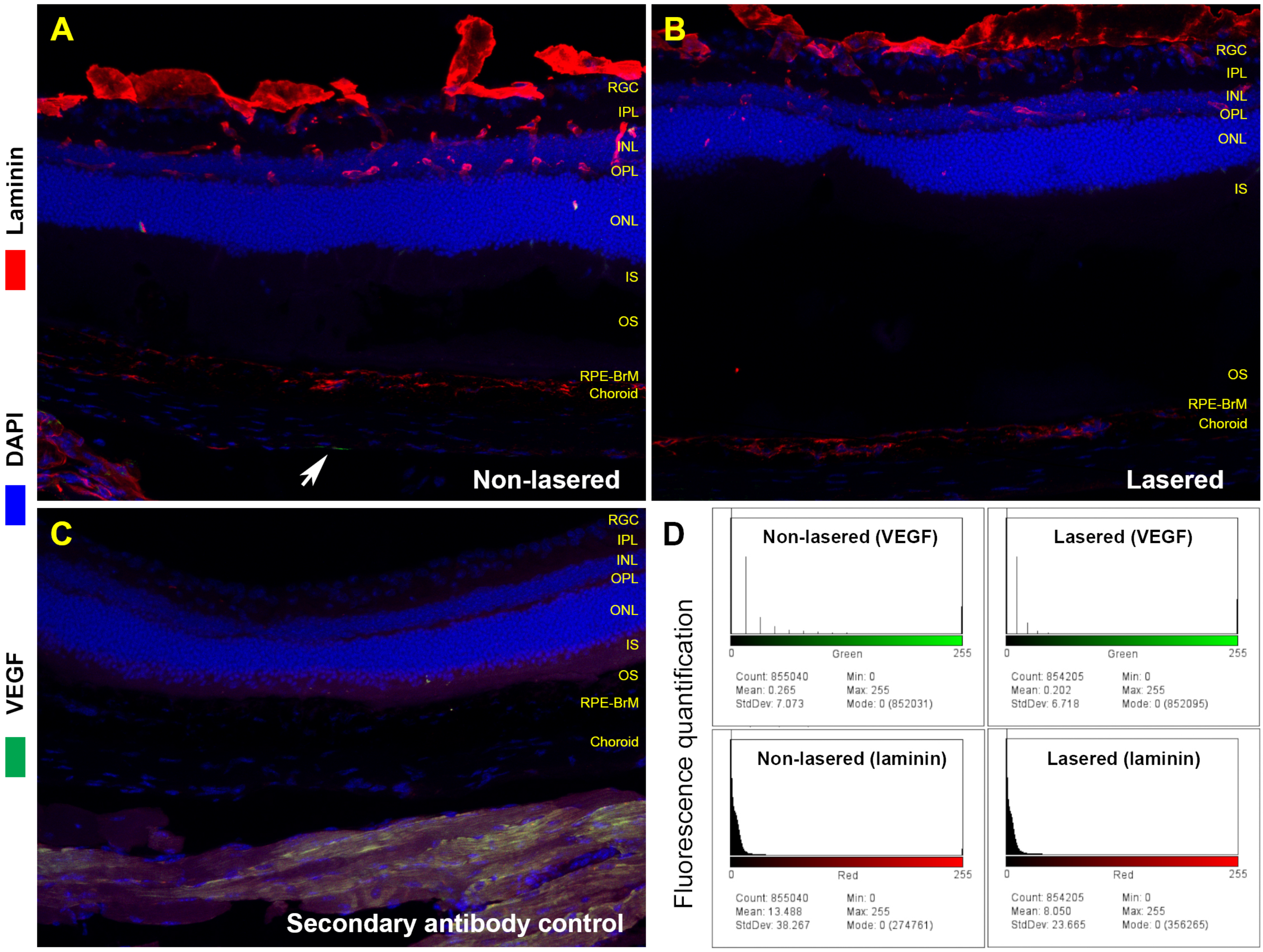

Figure S6: Comparison of VEGF levels in lasered mouse retinas after 4 weeks. The expression of vascular endothelial growth factor (VEGF), an indicator of angiogenesis and choroidal neovascularisation (green, and indicated by an arrow) and the blood vessel marker laminin (red) was assessed between [A] non-lasered and [B] lasered areas in mouse retinas. Although some detachment of the retina had occurred during tissue processing, GA-like lesions could be identified by the presence of a collapsed retinal layer. We found no evidence of VEGF upregulation in lasered sites above baseline expression in nearby non-lasered tissues. Laminin staining indicate retinal/choroidal vessels, which also showed no evidence of any neovascularisation at lasered sites. [C] A confocal-immunofluorescence image of secondary antibody only control tissue is shown alongside. Nuclei were stained with DAPI (blue). [D] We also measured the fluorescence levels of VEGF and laminin in non-lasered and lasered sites. The x axis represents fluorescence values and the y axis shows the number of pixels for each channel. The total pixel counts for each channel (VEGF: green and Laminin: red) in the whole field is shown as mean, modal, minimum and maximum fluorescence values. This quantification confirmed lack of VEGF upregulation or increased laminin expression (vessel proliferation) in lasered sites compared to adjacent non-lasered tissues in the GA-like mouse model.

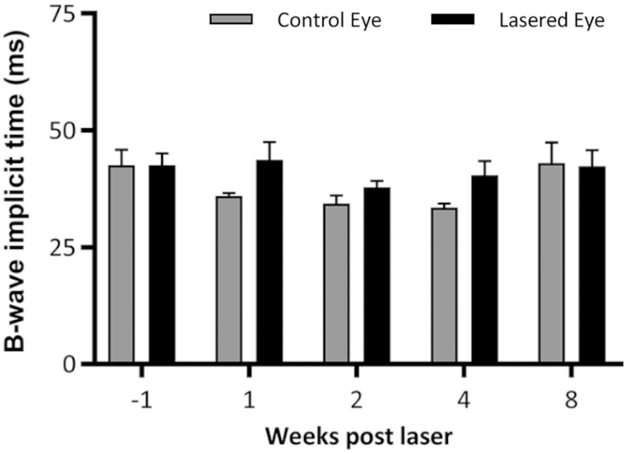

Figure S7: B-wave implicit times show no change between successive weeks in lasered eyes. Data analysed using two-way ANOVA followed by Holm-Sidak post-hoc testing.

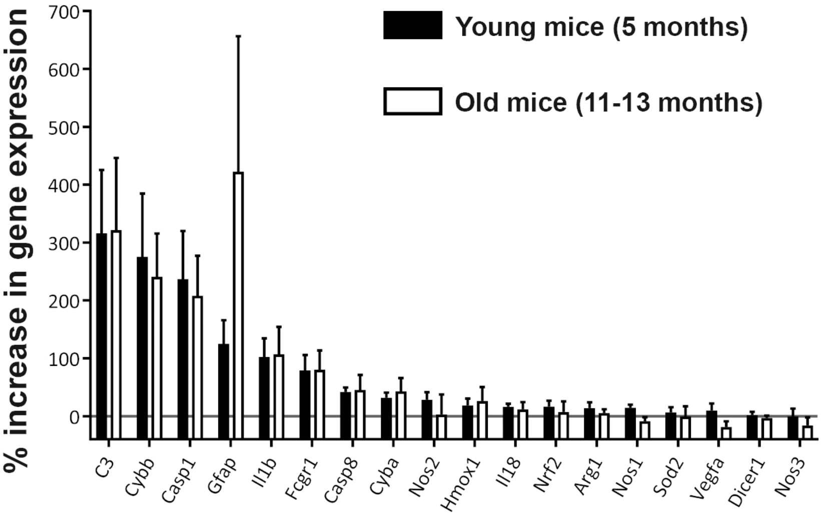

Figure S8: Comparison of mRNA profiles between young and old lasered eyes showed no significant differences. Data analysed using multiple paired one-tailed t-tests followed by Holm-Sidak multiple comparisons correction.

	Minimum	Maximum	Optimum
Primer length (bp)	15	25	20
Melting temperature (^{0}C)	57	63	60
Max 5' self-complementarity	0	7	0
Max 3' self-complementarity	0	3	3
GC content (%)	35	65	50
PCR product size (bp)	70	300	-

Table S1: Summary of primer conditions. Criteria used during primer design using Primer-BLAST (NCBI). Where possible, one primer of each pair recognised an exon-exon boundary to prevent the amplification of genomic DNA.

Gene	Gene ID	Common aliases	Fwd 5'-3'	Rev 5'-3'	Efficiency
Arg1	11846		ACAAGACAGGGCTCCTTTCAG	CTTGGGAGGAGAAGGCGTTT	99%
C3	12266		CAACAACCAACACGGCATCT	AACTGGGCAGCACGTATTCC	95%
Casp1	12362		AGGCACGGGACCTATGTGAT	AGCTGATGGAGCTGATTGAAG	119%
Casp8	12370		AGCACAGAGAGAAGAATGAGCC	TTGGCGAGTCACACAGTTCC	103%
Cyba	13057	p22phox	TGGCCTGATTCTCATCACTGG	TAGAGTAGGCGCCGAAATACC	87%
Cybb	13058	Nox2, gp91phox	GCTGGAAACCCTCCTATGACTT	GCCAAAACCGAACCAACCTC	98%
Dicer1	192119		ACATGACGAGGAGGAGACCA	ACTCTGGAATTGCTTTGGGGT	87%
Fcgr1	14129	CD64, FcγRI	TACTTTGGGTTCCAGTCGGT	CCTGTATTCGCCACTGTCCT	103%
Gapdh	14433		TGAACGGGAAGCTCACTGG	TCCACCACCCTGTTGCTGTA	115%
Gfap	14580		AGTGGTATCGGTCTAAGTTTGC	GACTCCAGATCGCAGGTCAA	104%
Hmox1	15368	HO-1	GCTAGCCTGGTGCAAGATAC	TGGGGGCCAGTATTGCATTT	112%
Il1b	16176		CAAAAGATGAAGGGCTGCTTCC	ATGTGCTGCTGCGAGATTTG	141%
Il18	16173		TCAAAGTGCCAGTGAACCC	GTCACAGCCAGTCCTCTTACT	110%
Nfe2l2	18024	NRF2	GGTTGCCCACATTCCCAAAC	GCAAGCGACTCATGGTCATC	100%
Nos1	18125	nNOS	AGAGGAAGAGCTACAAGGTCC	GGCCGAAGACTGAGAACCTC	90%
Nos2	18126	iNOS	ATGCCACCAACAATGGCAAC	TAGGTCGATGCACAACTGGG	103%
Nos3	18127	eNOS	CCGGAGAATGGAGAGAGCTT	CAGAAGTGGGGGTATGCTCG	115%
Sod2	20656	MnSOD	GAACAATCTCAACGCCACCG	CCAGCAACTCTCCTTTGGGTT	104%
Vegfa	22339		GATCCGCAGACGTGTAAATGTT	TCACCGCCTCGGCTTGTCACAT	99%

Table S3: Efficiencies of primers used for mRNA analysis. 1:5 serial dilution of retinal cDNA (neat to 1:625) run with individual primers and expression measured by qPCR under the same conditions as descibed in methods. Data analysed by plotting on a linear scale with log (DNA copy number) on the x axis and threshold Ct value on the y axis. Slope of the linear trendline was calculated and used to establish efficiency using the following equation: $E = (-10^{(-1/slope)}-1)*100$

Gene	Gene ID	Common aliases	Forward primer 5'-3'	Reverse primer 5'-3'
Arg1	11846		ACAAGACAGGGCTCCTTTCAG	CTTGGGAGGAGAAGGCGTTT
C3	12266		CAACAACCAACACGGCATCT	AACTGGGCAGCACGTATTCC
Casp-1	12362		AGGCACGGGACCTATGTGAT	AGCTGATGGAGCTGATTGAAG
Casp-8	12370		AGCACAGAGAGAAGAATGAGCC	TTGGCGAGTCACACAGTTCC
Cyb-a	13057	p22phox	TGGCCTGATTCTCATCACTGG	TAGAGTAGGCGCCGAAATACC
Cyb-b	13058	Nox2, gp91phox	GCTGGAAACCCTCCTATGACTT	GCCAAAACCGAACCAACCTC
Dicer1	192119		ACATGACGAGGAGGAGACCA	ACTCTGGAATTGCTTTGGGGT
FcγRI	14129	CD64, FcγRI	TACTTTGGGTTCCAGTCGGT	CCTGTATTCGCCACTGTCCT
GAPDH	14433		TGAACGGGAAGCTCACTGG	TCCACCACCCTGTTGCTGTA
GFAP	14580		AGTGGTATCGGTCTAAGTTTGC	GACTCCAGATCGCAGGTCAA
Hmox1	15368	HO-1	GCTAGCCTGGTGCAAGATAC	TGGGGGCCAGTATTGCATTT
IL-1β	16176		CAAAAGATGAAGGGCTGCTTCC	ATGTGCTGCTGCGAGATTTG
IL-18	16173		TCAAAGTGCCAGTGAACCC	GTCACAGCCAGTCCTCTTACT
Nfe2l2	18024	NRF2	GGTTGCCCACATTCCCAAAC	GCAAGCGACTCATGGTCATC
Nos1	18125	nNOS	AGAGGAAGAGCTACAAGGTCC	GGCCGAAGACTGAGAACCTC
Nos2	18126	iNOS	ATGCCACCAACAATGGCAAC	TAGGTCGATGCACAACTGGG
Nos3	18127	eNOS	CCGGAGAATGGAGAGAGCTT	CAGAAGTGGGGGTATGCTCG
SOD2	20656	MnSOD	GAACAATCTCAACGCCACCG	CCAGCAACTCTCCTTTGGGTT
VEGF-A	22339		GATCCGCAGACGTGTAAATGTT	TCACCGCCTCGGCTTGTCACAT

Table S2: List of primers used for mRNA analysis.
